# Diphtheria

**Published:** 1899-10-07

**Authors:** P. Watson Williams

**Affiliations:** Physician to Clifton College; and Physician in Charge of the Department for Diseases of the Throat; Bristol Royal Infirmary.


### DIPHTHERIA.

By P. Watson Williams, M.D.Lond., Physician to Clifton College; and Physician in Charge of the Department for Diseases of the Throat; Bristol Royal Infirmary.

(*Continued from page 457, Vol. xxvi.*)

**Bacteriology.**—The diphtheria bacillus is extremely polymorphic, and presents many variations in size and shape—now long and curved, now short and straight, one time clubbed at the end, another swollen at the centre with pointed ends, Thus, while nearly always presenting characteristics sufficiently marked to enable an expert to differentiate it from other micro-organisms, its identification may sometimes cause considerable difficulty, and the difficulties are increased by the close resemblance of certain other non-diphtherial bacilli.

The bacilli contain no spores, have no flagellæ, and are immobile. They always arrange themselves in more or less parallel groups, or in clusters at various angles, and they never form chains. Two varieties, may be described, the long and the short pathogenic: (1) The long form, sometimes called "typical" diphtheria bacilli, are straight, slightly curved or ʃ-shaped segmented rods, generally clubbed at one or both ends, of varying length and thickness, and often grouped in parallel bundles of two or more. The ends, if not clubbed, are rounded. When stained they have a granular or segmented appearance from taking up the dye more deeply in parts, and when only one or two segments of a bacillus are stained "polar staining," the "granules" correspond with the ends of the bacillus. (2) The short form generally occurs as short, slightly curved, or straight rods, in parallel groups of two or more, slightly swollen and deeply stained at one end, the other end tapering; or swollen in the middle, which is deeply stained, and pointed at the ends. Some of the bacilli with deeply-stained centre are diplo-bacilli—two bacilli still united end to end. Others are somewhat pear shaped, the "sheath bacilli" of Peters. The division of these bacilli into two groups is more or less arbitrary, as transitional forms will often be found in a culture which mainly yields the one or other of these groups. Nor can any reliance be placed in the view that the short form is less virulent than the long, though the pear-shaped variety is more usually an involution form. Kanthack[10] states "that after an extensive examination he can say confidently that it is futile to base a prognosis on the type of organism present." Some of the worst cases he has seen were associated with the short variety exclusively.

From experimental investigations on the toxin formation during the growth of the bacillus diphtheriæ, Kossel[11] found that the maximum toxicity of a culture is reached in five days.

**Bacilli Resembling the Klebs-Loeffler Bacillus** (so-called pseudo-diphtheria bacilli).—Certain bacilli which closely resemble the diphtheria bacillus are found not only in association with the Klebs-Loeffler bacillus, but in the normal secretions of the mouth, and on the conjunctiva in health, and in the disease xerosis conjunctivæ. By some the bacillus of xerosis is considered identical with Von Hoffmann's bacillus, by others it is believed to be a distinct micro-organism.

*Hoffmann's Bacillus* has been the subject of much discussion. In a twenty-four hours' culture on agar or serum it appears as a short pyramidal bacillus arranged in pairs, set end to end in parallel groups of two or more, that is to say, it is a diplo-bacillus. Its protoplasm stains more deeply at the opposed ends. It occurs in many forms of sore throat, and sometimes in association with the long or short forms of the true Klebs-Loeffler bacillus, but it is nonpathogenetic to guinea pigs, or, at most, causes a local inflammation at the site of injection.

Bacilli morphologically indistinguishable from the Klebs-Loeffler bacillus, but nonpathogenic to guinea-pigs, are regarded by some as a distinct form. Roux, Yersin, and E. Fraenkel believe that this pseudo-diphtheritic bacillus is simply an attenuated form of the Klebs-Loeffler bacillus, which under proper conditions may regain its virulence. Escherich and the German and English school generally regard this bacillus as a distinct microbe. Hewlett and Knight state that they were sometimes able to convert the non-virulent pseudo-diphtheria bacillus into a virulent Klebs-Loeffler bacillus; and it has apparently been possible by careful treatment to convert a typical Klebs-Loeffler bacillus into a typical non-virulent pseudo-diphtheria bacillus. It, therefore, seems hardly safe to assume that non-virulent bacilli, morphologically identical with the Klebs-Loeffler bacillus, is not potentially a true bacillus diphtheriæ, but inasmuch as bacilli non-virulent to guinea-pigs, but morphologically resembling the bacillus diphtheriæ, has frequently been isolated from noma, cancrum oris, impetigo, various chronic ulcerations in numerous healthy throats, and by Cantley[12] in ordinary acute febrile nasal catarrh (bacillus coryzæ segmentosus), it is hardly practicable to regard the presence of such non-virulent bacilli as a sufficient reason for isolation. Points of distinction between the true and the pseudo-diphtheria bacillus, which may eventially prove reliable, are referred to in connection with the Neisser test.

The proportion of examinations yielding pure and mixed cultures, and the different micro-organisms that are frequently present is fairly shown by the tabulated notes on 600 cases, forwarded from various practitioners and medical officers of health to the British Institute for diagnosis, reported by Hewlett and Nolan[13] here appended:—

| The following organisms were present alone or associated with the B. Diphtheriæ. | Cases in which the Diphtheria bacillus was present, alone or associated with other organisms. | Cases in which the Diphtheria bacillus was absent. |
|---|---|---|
| Bacillus diphtheriæ alone | 216 | Pseudo-diphtheria 2 |
| Streptococci | 6 | 32 |
| Micrococci | 55 | 79 |
| Bacilli | 19 | 41 |
| Torulæ | 9 | 1 |
| Sarcinæ | 6 | 2 |
| Streptococci and micrococci | 2 | 23 |
| Micrococci and bacilli | 9 | 19 |
| Streptococci and bacilli | 1 | 5 |
| Torulæ and bacilli | 1 | 3 |
| Sarcinæ and bacilli | 0 | 3 |
| Micrococci (including streptococci) and sarcinæ | 6 | 8 |
| Micrococci (including streptococci) and torulæ | 4 | 14 |
| Many forms present together | 19 | |
| | 353 | 247 |

These results are only approximate, as they are based on the more or less brief examination, necessary to determine the presence or absence of the diphtheria bacillus, and no special pains were taken to observe all the organisms which might be present.

It is noteworthy that these were practically pure cultures of the bacillus diphtheriæ in 216 out of the 353 cases in which it was found.

**Detection of the Bacillus.**—*Direct Examination.*—A diagnosis can sometimes be made by the direct examination of a cover-glass film, and, pending the more reliable result obtainable from the examination of artificial cultures, is worthy of trial. The staining of the film should be made very shortly after taking the secretion or membrane from the throat, as saprophytic organisms rapidly multiply, and crowd out the diphtheria bacilli. The diphtheria bacilli may be stained by methylene-blue or carbol-fuchsine, and they are not decolourised by Gram's method.

*Examination of Cultures.*—Artificial cultures may be made on serum, agar, serum-agar, glycerine agar, gelatine, glucose bouillon, litmus milk, or litmus whey. Kanthack and Stephens[14] recommend an easy method of preparing serum-agar in less than an hour. To every 100 c.cm. of serous exudation—ascitic, pleuritic, or hydrocele fluid—2 per cent. of a 10 per cent. solution of caustic potash must be added. To this they add 1·5 to 2 per cent. of agar-agar, previously soaked in acidulated water, and then boil the mixture in a Koch's steamer till the agar-agar is well dissolved. It is then filtered through a hot-water funnel, and to the filtrate 5 per cent. of glycerine and 1 per cent. of grape sugar should be added. It may then be poured into test tubes, and after sterilisation it will set firmly and be ready for use.

Among the advantages that are claimed for this serum agar-agar are the following: (1) It is quickly prepared, easily obtainable, and cheap; (2) its selective action on the diphtheria bacillus is greater than that of any other serum preparation known to its authors; its inhibitory action on staphylococci, bacillus coli communis, &c., is extraordinary.

The culture tube may be inoculated from a piece of membrane if that be obtainable, first washing the membrane several times in sterile normal salt solution, and picking off a small piece with a platinum needle (first sterilised by holding it in a flame till it is red-hot, and then letting it cool), or the platinum needle may be plunged into the substance of the membrane. Or a sterilised needle or swab may be passed over the surface of the membrane in the throat, then, withdrawing the wool plug of the culture tube, the needle or swab is lightly drawn over the surface of the culture medium in the tube without breaking the surface and the tube closed with the wool, which must not have been laid down, or it will have become impregnated with extraneous micro-organisms.

If no membrane is visible, or if the case is presumably a laryngeal diphtheria, the secretions from the fauces or the back of the pharynx should be examined —not that from the tonsillar surface.

Suspected milk or urine, &c., should be diluted, and then centrifugalised, cultures, or inoculations into guinea-pigs, being made from the sediment. The effect of dilution of the fluid is to lower its specific gravity, so that the bacilli tend to fall, instead of permeating the fluid indifferently.

The culture tube having been inoculated is then incubated at a uniform temperature of 38·0 to 38·5 deg. Cent. In from 12 to 18 hours it is ready for examination.

The macroscopical appearance of the growth is usually fairly characteristic. The Klebs-Loeffler bacilli at first form small, round, discrete, grayish-white spots, with a thicker and more opaque and slightly yellow centre; but the translucent spots by extension coalesce, and come to form an opaque patch, with a thick and prominent centre.

The bacilli may be stained by Gram's method with Loeffler's methylene-blue, but Max Neisser's double stain is probably the most certain means of differentiating the diphtheria bacillus. Leedham-Green,[15] who introduced the method in this country, speaking from a long experience, insists on the necessity for adhering strictly to Neisser's directions, viz., that the culture be grown in solidified blood serum at a temperature of 35 deg. Cent. (certainly not higher than 36 deg. Cent.), and that the growth should be examined when from 10 to 20 hours old. The bacilli are treated first with a dilute acid solution of methylene-blue, and subsequently with Bismarck brown.

The stains must be freshly prepared, or the blue quickly loses its power. If examined too early the staining may be limited to only a few bacilli; on the other hand, if older than 20 hours, other bacilli beside the diphtheria bacillus may show a somewhat similar staining. Symes, of Bristol, my colleague at the Bristol Royal Infirmary, also speaks enthusiastically of the value, rapidity, and trustworthiness of this method. The polar granules take up the blue, and appear as dark blue or almost black dots, more oval than round, at one or both ends of the bacillus. Occasionally there is a third situated towards the middle and sometimes a fourth is seen.

Baginsky,[16] C. Fraenkel,[17] and others consider the test a reliable means of differentiating between the bacillus diphtheriæ and the pseudo-bacillus, as the latter does not show any staining of polar granules, but Leedham-Green does not think that this double stain is accurate enough to decide once and for all between the true and the false varieties of bacilli. He believes that the polar granules are the result of a degenerative process, and he regards the positive reaction as of greater value than the negative. If the suspicious micro-organism stains with characteristic polar granules, then it is certainly diphtheria; if, on the other hand, no polar granules are present, then most probably it is not diphtheria. Finally, Leedham-Green states that for the purposes of diagnosis the Neisser stain should be supplemented by an examination of "the hanging drop" and staining by Gram's method.

The production of acid by the bacillus when grown in broth generally, though not always, differentiates the true from the false bacillus. Both forms flourish in antitoxic serum. The result of inoculation of guinea-pigs, when available, serves to demonstrate attenuated virulence, even if it cannot be said to afford definite evidence that one is not dealing with the Klebs-Loeffler bacillus; it is, therefore, often of great value from a clinical standpoint.

[10] Allbutt's System, Vol. I. [11] Cent. f. Bakt., 1896, No. xix., p. 977; Brit. Med. Jour., Dec. 19, 1897. [12] Report on Etiology of Influenza Cold; Twenty-fourth Annual Report of Local Government Board, 1894-1895, supplement, p. 455. [13] Brit. Med. Jour., Feb. 1. 1896. [14] Lancet, Mar. 28, 1896. [15] Birm. Med. Rev., Oct., 1898. [16] Diph. und Diphtherisch Croup, 1898; Brit. Med. Jour., Dec. 3, 1898. [17] Berl. Klin. Woch., Dec. 13, 1897; Epit. Brit. Med. Jour., Jan. 22, 1898.

